# Genetic adaptations shaping survival, pregnancy, and life at high altitude and sea level

**DOI:** 10.1098/rstb.2024.0170

**Published:** 2025-08-21

**Authors:** Wanjun Gu, Elijah S. Lawrence, A. Mark Evans, Tatum S. Simonson

**Affiliations:** ^1^ Department of Medicine, Division of Pulmonary, Crtiical Care, Sleep Medicine, and Physiology, University of California, San Diego, CA, USA; ^2^ Department of Neurology, Weill Institute for Neurosciences, University of California, San Francisco, CA, USA; ^3^ Department of Biomedical Engineering, Duke University, Durham, NC 27708, USA; ^4^ Institute for Neuroscience and Cardiovascular Research, University of Edinburgh, Edinburgh, UK

**Keywords:** altitude, hypoxia, adaptation, pregnancy

## Abstract

Advancements in genetic research have greatly enhanced our understanding of human adaptation to high-altitude environments through the identification of genetic markers linked to hypoxia tolerance. Our recent studies identify key genes associated with haematological and ventilatory traits in Andeans. Adaptive variation at *EPAS1*, encoding endothelial PAS domain protein 1, a key regulator in the hypoxia-inducible factor (HIF) pathway (the alpha subunit of HIF2), has been associated with relatively low haematocrit at high altitude, which may be linked directly or indirectly to improvements in oxygen transport and/or delivery, while *PRKAA1*, encoding the AMP-activated protein kinase (AMPK) alpha-1 subunit, has been linked to ventilatory responses during wakefulness that are further associated with sleep phenotypes with metabolic implications. The relevance of these genetic adaptations extends beyond adult physiology; e.g. other studies have associated an adaptive genetic signature at *PRKAA1* with pregnancy outcomes in Andean populations. Understanding how adaptive genetic variations in *EPAS1* and *PRKAA1* contribute to hypoxia tolerance offers a foundation for investigating broader evolutionary mechanisms of high-altitude adaptation, particularly in the contexts of pregnancy and fetal development, where oxygen availability is crucial. Integrative studies that combine molecular, physiological, and evolutionary perspectives offer promise in revealing the complexities of high-altitude adaptation and its relevance to hypoxia-related health challenges in both highland and lowland populations.

This article is part of the discussion meeting issue ‘Pregnancy at high altitude: the challenge of hypoxia’.

## 1. Introduction

**Figure 1.**
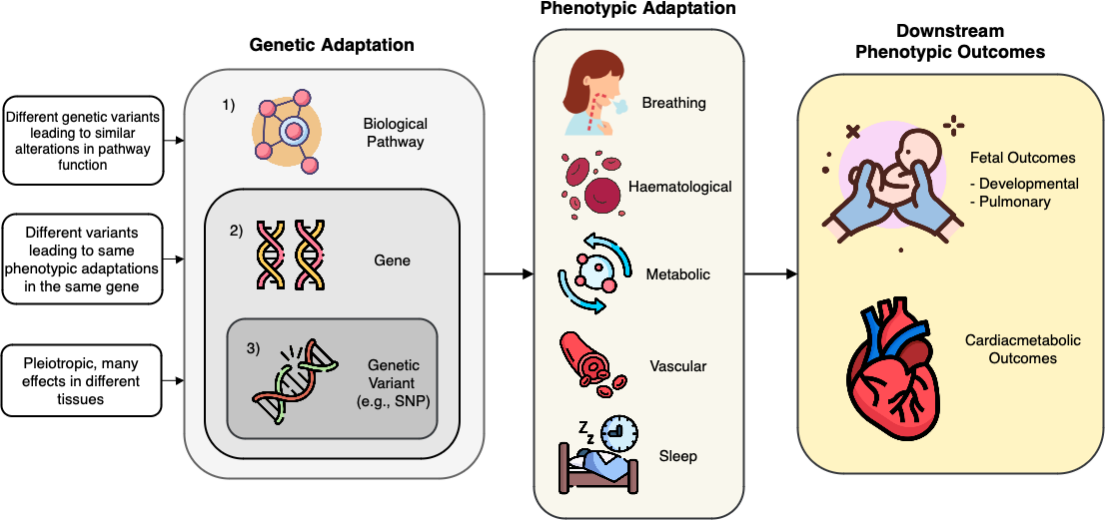
Levels of genetic adaptation, phenotypes and outcomes. Genetic adaptation acts at various levels, including 1) biological pathways, whereby changes may arise owing to compensatory mechanisms or convergent evolution, allowing for adaptation at a systemic level rather than a single-gene level; 2) gene, as the same adaptive outcome is associated via independent genetic changes in the same locus; and 3) variants with potential pleiotropic effects, where a particular variant, such as a single nucleotide variant (SNP), can impact multiple traits across different biological processes, resulting in diverse phenotypic outcomes that may be directly or indirectly subject to selection.

Advancements in genetic research have greatly enhanced our understanding of how humans adapt to high-altitude environments. This progress is primarily owing to the development of high-throughput genotyping and powerful genome-wide tests that identify positive selection that has occurred throughout hundreds of generations in humans [1] and help prioritize functional adaptive genetic markers [2]. In various studies of high-altitude adaptation, these genomic regions have included canonical hypoxia-inducible factor (HIF) and non-HIF pathways that are crucial for adaptation to low oxygen (O_2_) availability in both humans [3,4] and other highland species [5,6], yet in many cases, direct links to phenotypes are unknown. Our most recent studies have focused specifically on genetic variants within *Endothelial PAS Domain Protein 1*, *EPAS1,* and *AMP-Activated Protein Kinase Alpha-1 *(*AMPK-a1*), *PRKAA1*, respectively. While many other genes show evidence of selection for high altitude, *EPAS1* and *PRKAA1* are the focus of this review owing to their established roles as critical to high-altitude adaptation and relevance to various high-altitude-related phenotypes, including those described in pregnancy [7–13]. Genetic adaptations examined at different biological scales, whether at the variant, gene, or pathway level (figure 1), highlight the intricate and multifaceted nature of hypoxia responses and the potential for adaptation to have direct, indirect, and interconnected phenotypic effects. In this review, *EPAS1* and *PRKAA1* serve as representative examples of the broader pathways involved in O_2_ sensing, metabolic regulation, and physiological adaptation to hypoxia.

*EPAS1*, a key player in the HIF pathway, has been identified as a top signal of selection [14–16] and linked to haemoglobin concentration ([Hb]) in individuals of Tibetan ancestry living at high altitude [15,16]. Similarly, our recent work has identified that more copies of the adaptive *EPAS1* variant are associated with lower haematocrit (Hct) in male Andean highlanders [10]. This variant (rs570553380 A>G p.[His194Arg]) is present at a moderate frequency in Andeans [10–12] and nearly absent in other human populations and nearly 100 vertebrate species [10]. While a relatively low [Hb] or Hct is observed in many Tibetan and some Andean highlanders, the underlying genetic mechanisms probably differ owing to distinct non-coding and coding variants identified, respectively, in each group. Since excessively high [Hb] can hinder fetal development across altitudes [17,18], and survival at birth is essential for passing on genes, maintaining a relatively low [Hb] may offer a crucial evolutionary advantage. Therefore, investigating how adaptive *EPAS1* variants impact pathway expression and whether these associations are detected with maternal [Hb] and influence pregnancy and reproductive outcomes in high-altitude hypoxic conditions is an important and ongoing area of research [13].

In other recent work, we have confirmed an adaptive genomic signature in Andeans previously reported at *PRKAA1* [19], which encodes the AMPK-α1 subunit, a cellular energy sensor that not only coordinates cell autonomous metabolic homeostasis but also phosphorylates and regulates transcriptional co-regulators, which in turn adjust gene expression programmes that influence developmental and adaptive metabolic control mechanisms. While we further identified associations with increased breathing responses to hypoxia and improvement in sleep phenotypes in Andeans [20], separate studies identified tagging variants in this same gene region associated with birthweight and intrauterine artery diameter in high-altitude pregnant Bolivian women [9]. The association between *PRKAA1* and breathing and/or sleep O_2_ saturation during pregnancy, when O_2_ fluctuations could impact fetal outcomes, remains to be examined. Nevertheless, indirect evidence suggests that the putatively adaptive *PRKAA1* variant may support perinatal lung development in highland Andeans, which can be impeded by maternal hypoxia or the hypertensive disorder of pregnancy, pre-eclampsia, at altitude [21] and also by AMPK deficiency in mice [22]. Additionally, the adaptive genomic signature in *PRKAA1* could conceivably promote apoptosis in mature erythroblasts [23], and thus offer a further adaptive mechanism for maintaining a relatively low Hct in some highland Andean populations, as suggested for other potentially adaptive genes important for high-altitude adaptation [24,25]. Evidence also suggests that AMPK-α1 promotes vasodilation of systemic vascular beds via both endothelium-dependent [26,27] and endothelium-independent mechanisms [28,29] to match tissue perfusion to local metabolic demand, e.g. skeletal muscle perfusion and functional hyperaemia in the brain.

While many studies focus on genetic adaptations, fewer have examined associations with physiological links, and those that have largely focused on haematological, ventilatory, vascular, and metabolic adaptations in adult men and, to a lesser extent, women. The impact of these traits probably extends to *in utero* and early life stages, which are critical periods for natural selection. Therefore, connections identified thus far may reveal residual or foundational phenotypes shaped by selection pressures, offering valuable clues into the direct genetic targets driving high-altitude adaptation. Amid the hundreds of genes identified in high-altitude adaptation studies, this review highlights two selection-nominated candidates recently examined for their roles in haematological and ventilatory responses in Andeans: *EPAS1* and *PRKAA1*. These genes provide a valuable framework for understanding how selection operates, i.e. through individual genetic variants, across genes in different populations, and within pathways that may function in parallel, converge, or complement one another (figure 1). While many candidate genes merit further exploration with additional physiological and molecular analyses [4,13,24], we highlight recent insights into these targets, their unique evolutionary processes, reported associations, and potential links to pregnancy outcomes.

## 2. Hypoxia-inducible factor pathway gene, *EPAS1*

*EPAS1*, which encodes the alpha subunit of HIF-2, is one of the top selection-nominated genes identified in humans [4,24,30,31] and other highland species [5,6]. HIF-2α is hydroxylated under normoxic conditions in an O_2_-dependent manner and targeted for degradation. Under hypoxic conditions, HIF-2α accumulates and localizes to the nucleus where it dimerises with its binding partner HIF-1β and acts as a transcription factor that influences the expression of hundreds of genes involved with physiological processes such as erythropoiesis, angiogenesis, metabolic regulation, and fetal development [32]. Tibetans and Andeans exhibit strong adaptive signals at various HIF and HIF-targeted genes [30] that appear to have unique functions, reflecting distinct evolutionary histories that, in some cases, converge in terms of phenotypic effects [10].

A key feature of hypoxia acclimatization is increased Hct, which varies significantly across and also within continental highland groups [33–35]. At high altitudes, increased Hct is typically sustained by erythropoietin, which boosts red blood cell production in response to hypoxia. This elevation in Hct enhances arterial O_2_ content and has been traditionally seen as an adaptive response to improve O_2_ delivery to tissues. While some Andeans exhibit elevated and even excessively high Hct [36], Tibetans living at altitudes of 4000 m or higher typically exhibit [Hb], directly proportional to Hct, within the range expected for people living at sea level [33,37–39]. The adaptive importance of this pattern became even more evident when population genomic studies uncovered strong evidence of positive selection in the *EPAS1* gene region [14–16], which was associated with relatively low (i.e., non-elevated) [Hb] in Tibetans at high altitude [15,16]. Interestingly, this *EPAS1* region introgressed from an archaic Denisovan population [40–42], providing a notable source of genetic variation that proved adaptive in Tibetans. Further studies have emphasized that variations in [Hb] may be influenced by physiological mechanisms beyond erythropoiesis, such as increased plasma volume [43].

**Figure 2.**
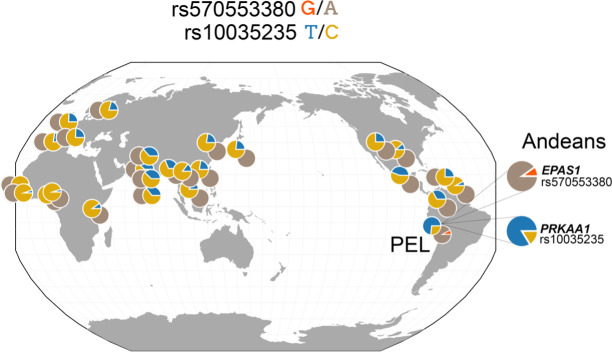
Global allele frequency distribution of *EPAS1* and *PRKAA1* variants associated with high-altitude adaptation in Andeans. Geographical distribution of allele frequencies for two key genetic variants: rs570553380 in *EPAS1* (G/A) and rs10035235 in *PRKAA1* (T/C). Pie charts represent the proportion of ancestral and derived alleles across various global populations. The *EPAS1* ‘G’ variant is found exclusively in Peruvians from Lima (PEL) and Andean populations, with a slightly higher frequency in the Andean high-altitude cohort. By contrast, the *PRKAA1* ‘T’ allele is observed globally but reaches its highest frequency in Peruvians, with an even greater prevalence in Andean highlanders.

We recently examined a protein-coding missense variant rs570553380 A>G p.[His194Arg] within the sixth exon of *EPAS1* exhibiting a signal of selection in data from Quechua Andeans with a wide range of Hct studied at 4340 m [10]. This specific variant, which is largely unique to Andean populations (0.2% in worldwide human populations from whom whole genome sequence (WGS) data have been generated; figure 2), was previously shown to be under early-stage positive selection in Colla highlanders in Argentina [11] and exhibits extended haplotype homozygosity in WGS data from 40 Andeans [10]. The putatively adaptive haplotype, characterized by the presence of rs570553380(G), is observed at low frequencies (7–9%) in both the Peruvians from Lima (PEL) and Andean populations and is almost completely absent in non-Andean global populations [10].

Similar to links between tagging single nucleotide variants at the *EPAS1* locus in Tibetans, our genotype–phenotype studies provide, to our knowledge, the first evidence for a negative association between an *EPAS1* variant and Hct in Andeans (*n* = 224) [10]. These findings highlight that Andean and Tibetan hypoxia tolerance may directly or indirectly impact phenotypic traits such as Hct via different alterations within the same gene. Despite suggestive evidence for convergent changes in Hct mediated by *EPAS1*, the molecular mechanisms of action appear distinct between continental groups: the potentially causative changes involve *cis*-regulatory mutations in Tibetans [44,45] and a coding change in *EPAS1* of Andeans [10–12].

Using advanced gene-editing techniques, we have observed changes in gene expression related to low O_2_ in human cells with this putatively adaptive variant and provide further functional support based on metabolomic analyses. Human embryonic kidney 293 T cells with one copy of the putatively adaptive ‘G’ (Arg, R) allele demonstrated lower expression of canonical HIF-2 target genes (HIF-2α protein levels similar across genotypes under hypoxia). In particular, expression levels of *ADM, BHLE40, HILPDA, ING4,* and *VEGF* were significantly decreased in cells heterozygous for the *EPAS1* variant relative to wild-type cells from 12 to 24 hours of hypoxia exposure relative to normoxia [10]. The observed downregulation of canonical HIF targets in cells with the Andean-specific missense variant parallels the loss-of-function phenotype similarly observed in studies of Tibetan-specific non-coding variants in *EPAS1* [11,45,46].

Integration of omics data can also help reveal causal relationships. Metabolomics data from 115 Andeans identified 298 metabolites associated with Hct (false discovery rate <0.05) [10]. Cross-comparison with the large publicly available FINRISK cohort (*n* = 1170) validated 12 of these metabolites, including an endogenous cannabinoid arachidonoyl ethanolamide, which was linked to higher Hct. Among them, three unknown metabolites were also associated with rs570553380 in Andeans, negatively correlating with the adaptive ‘G’ allele and positively with Hct. Mendelian randomization suggests these metabolites are downstream of red blood cell production. Future research could explore metabolomic associations beyond Hct to uncover associations with key phenotypic traits and further clarify the connections between genotype, metabolites, and physiological outcomes.

While these findings provide insights into altered gene expression and omics profiles resulting from this specific missense variant, previous *EPAS1* loss-of-function studies in animal models suggest a protective effect against chronic hypoxia. Heterozygous knockout mice *Epas*1+/− exhibit lower [Hb] and reduced risk of pulmonary hypertension and right ventricular hypertrophy relative to wild-type mice following exposure to sustained hypoxia [47,48], with recent studies based on a Tibetan-specific variant demonstrating pleiotropic effects [44], i.e. the ability of a single gene to influence multiple distinct physiological traits (figure 1). Recent *in vivo* studies demonstrate the H194R allele is hypomorphic and protects against pulmonary arterial hypertension [12]. Additionally, high-altitude deer mice with a putatively adaptive *Epas1* variant exhibit reduced ventilatory chemosensitivity in chronic hypoxia, suggesting further importance of the control of breathing at altitude [49]. Future studies could examine additional *in vivo* effects during pregnancy and *in vitro* assessments in additional cell types, e.g. a human umbilical vein endothelial cell culture or organoid model that more closely resembles a vascularized organ, to represent the impact on global gene expression and nuances that could result in conditions of chronic hypoxia.

## 3. AMP-Activated Protein Kinase Catalytic Subunit Alpha-1, encoded by the *PRKAA1* gene

During the evolution of complex physiological systems selective pressures have led to successful processes being used in different ways where they were fit for purpose and this teleological argument was applied to AMPK [50], where a growing body of evidence now suggests its role has indeed been extended beyond cell autonomous metabolic homeostasis to serve a variety of cell- and tissue-specific functions, including the regulation of O_2_ and thus energy (ATP) supply to the body as a whole. Adapting to high-altitude hypoxia involves complex genetic and physiological changes to maintain O_2_ homeostasis, with reduced O_2_ availability triggering responses to optimize O_2_ saturation. One of the most crucial of these responses is the hypoxic ventilatory response (HVR), which is critical for short-term acclimatization and long-term adaptation to hypoxic conditions, leading to increased ventilation and improved O_2_ uptake. However, there is significant variability in the HVR between individuals and populations that underlies differences in how individuals adapt to high-altitude environments. In some cases, individuals exhibit a blunted HVR, which is observed more frequently in some Andeans and other populations with long-term high-altitude exposure, such as lowlanders of European and Chinese ancestry who move to altitude, and less in permanent residents with Tibetan ancestry [51–55].

Variation in the HVR is an important determinant of an individual’s short-term ability to acclimatize to hypoxia and may play a key role in adaptations that have occurred throughout the course of hundreds of generations in high-altitude environments. Knockout studies in mice reveal that deleting the gene (*Prkaa1*) encoding the catalytic α1-subunit of AMP-activated protein kinase in catecholaminergic neurons impairs the HVR and markedly increases apnoea duration and frequency during hypoxia [56,57]. Critically, the HVR is also impaired with increases in apnoea duration and frequency in mice with deletion in catecholaminergic neurons of the gene (*Stk11)* encoding Lkb1 [57], the upstream kinase that activates AMPK in response to metabolic stressors. By contrast, the HVR remains unaffected by global deletion of the gene (*Camkk2)* which encodes the calcium calmodulin-activated kinase kinase 2 (CaMKK2), and thus removal of calcium-dependent pathways to AMPK activation in mice [56], which can be augmented by energy stress through increases in AMP levels (reviewed in [58,59]). Thus, it is the canonical and ubiquitous energy-sensing pathway to AMPK activation that coordinates the HVR, probably downstream of mitochondria. As a universal energy sensor, AMPK detects drops in energy (AMP : ATP ratio) levels and initiates a phosphorylation cascade that regulates numerous downstream proteins involved in maintaining metabolic stability [59], including therein transcriptional co-regulators, such as histone deacetylases (HDAC) 4/5 [60] and ten–eleven translocation (TET) 2 [61,62], which in turn impact gene expression programmes that influence developmental and adaptive metabolic control mechanisms.

Although physiological hypoxia is known to activate AMPK by modifying AMP : ATP ratios and thus LKB1-dependent phosphorylation of AMPK in O_2_-sensing cells, the precise connection between AMPK and HIFs and the extent of tissue-specific effects remains unclear [63]. However, it is evident that HIF2α/EPAS1 may be important for the expression of nuclear-encoded mitochondrial subunits, such as cytochrome C oxidase subunit 4 isoform 2 (COX4I2), that may confer sensitivity to hypoxia within the physiological range [64,65] and may thus determine, at least in part, in which cells AMPK is activated during hypoxia. It is also evident that AMPK and HIFs regulate complementary adaptive pathways, if not each other, and may thereby indirectly influence each other and in a cell-specific manner. For example, both AMPK and HIF increase glucose uptake, glycolytic flux, and autophagy, and suppress protein translation. By contrast, in mitochondrial biogenesis they oppose each other, AMPK promoting mitochondrial biogenesis while HIF signalling may decrease mitochondrial mass. Therefore, AMPK and HIF may, each in part, deliver appropriate adjustments to metabolism in a cell- and thus system-specific manner (for detailed review see [66]).

Given its crucial role in breathing in animal models [56,57] and previous reports of adaptation in Andeans [9], we hypothesized that adaptive variation in *PRKAA1* could be further linked to beneficial breathing and sleep phenotypes at high altitude. We explored relationships specifically between the *PRKAA1* promoter variant rs10035235(T) (figure 2) and hypoxic and hypercapnic ventilatory responses as well as sleep phenotypes in high-altitude Andeans [20]. Globally, *PRKAA1* rs10035235 alleles segregate at relatively balanced frequencies, but the T allele is more frequent in the Peruvian population (PEL from 1000 genomes; figure 2). In the Andean highlander cohort, the T allele frequency is even higher, consistent with the hypothesis that standing genetic variation, which could be common worldwide, can rise in frequency under natural selection in a high-altitude environment.

Considering AMPK-α1 is crucial for the HVR and counteracts respiratory depression during hypoxia, the rs10035235(T) variant may enhance AMPK levels, potentially providing a selective advantage by strengthening ventilatory responses to hypoxia, especially during sleep and at high altitudes. Our findings suggest that rs10035235(T) could confer an advantage in hypoxic environments through increased *PRKAA1* expression, an augmented HVR, and higher O_2_ saturation during sleep in Andean men but not women. This may be significant because previous studies report a twofold higher hypoxic ventilatory response [67] and reduced pulmonary vascular resistance [68] in pregnant women at week 36 and weeks 36−38, respectively, relative to postpartum. It is therefore possible that the putatively adaptive *PRKAA1* single nucleotide variant underlies sex-specific hormonally regulated adaptations in women (perhaps pre-menopausal) that are particularly relevant during pregnancy, although this remains to be determined. This possibility is intriguing because AMPK expression is regulated by sex hormones and stress hormones [69,70].

Given rs10035235(T) was associated with breathing and sleep phenotypes in Andeans and is prevalent as a standing genetic variation in non-highland populations, we tested its relevance in large genome cohorts, where it was further associated with protection from maladaptive sleep phenotypes [20]. It remains to be determined if this variant reduces the risk of sleep-disordered breathing and nocturnal hypoxaemia during pregnancy, as these could be critical factors for maternal and ultimately fetal oxygenation at high altitudes. The role of this variant in developmental maturation of the hypoxic ventilatory network should also be given significant consideration, given that the integrated HVR of adult mice that lack *Prkaa1* in catecholaminergic neurons is markedly reminiscent of the HVR observed in neonates, i.e. marked late hypoxic ventilatory depression potentially culminating in aponeic episodes, along with preservation of hypercapnic hypoxic responses. The putatively adaptive *PRKAA1* promoter variant may also be important to neonatal lung development after birth and enhanced AMPK-α1 expression to adulthood, and could also explain the postnatal persistence of pulmonary hypertension in Andeans [71], given that AMPK-α1 facilitates hypoxic pulmonary vasoconstriction [72].

## 4. Implications in pregnancy

High-altitude environments (≥2500 m) serve as a natural setting to study human adaptation, particularly in pregnancy, where O_2_ supply is crucial for maternal and fetal health. Research during the past few decades has identified unique pregnancy-related traits distinguishing long-resident high-altitude Andeans and Tibetans from recently acclimatized individuals [73–79] and potential genetic links to O_2_ saturation [80] as well as haematological and cardiovascular factors in the reproductive success of ethnically Tibetan women [81]. Adaptive genetic signatures in *PRKAA1* and *EDNRA*, which encodes endothelin receptor A, have been linked to birth weight in high-altitude Andeans, with *PRKAA1* further associated with larger uterine artery diameters [9]. While high-altitude pregnancy is associated with risks like pre-eclampsia and fetal growth restriction, Andean women may have protective factors, including enhanced uteroplacental blood flow, higher antioxidant levels, and lower cortisol, which contribute to better pregnancy outcomes compared to non-native populations [13]. In this respect, it is also conceivable that the putatively adaptive *PRKAA1* variant could support, through increased AMPK-α1 expression, neonatal lung development after birth in highland Andeans, which is impeded by pre-eclampsia and hypoxia at altitude [21], in murine models by both maternal hypoxia [82], and *Prkaa1/2* deletion in smooth muscles of mice [22]. In the context of persistent pulmonary hypertension of the newborn, there is functional redundancy between *Prkaa1* and *Prkaa2*; nevertheless, *Prkaa1* could be the primary driver of pulmonary vascular and thus alveolar development after birth. Additionally, the involvement of AMPK-related pathways in lung development is supported by findings that gestational hypoxia significantly disrupts mTORC1 activation in the fetal lung, potentially stalling critical pulmonary vascular development and laying the groundwork for pulmonary vascular disease in later life [83].

Given excessive [Hb] can lead to overly viscous blood and increased risks of maternal hypertension, hypoxemia, and other complications [18,21], it is plausible relatively lower [Hb], comparable to general sea-level values and linked to genetic factors, would be beneficial in high-altitude environments. These and potentially other adaptive variants associated with [Hb] at high altitudes [4,84] may help limit excessive [Hb] in high-altitude pregnant women; e.g. some Tibetan women demonstrate higher uterine artery blood flow and higher birth weights compared to lowland (Han Chinese) women at the same altitude [74]. In addition to preventing severe polycythaemia, *EPAS1* adaptations may also lower the risk of pre-eclampsia, which is an often serious hypertensive complication in pregnancy exacerbated by hypoxia and reduced placental perfusion. Of note, healthy high-altitude pregnancies show a greater increase in erythropoietin compared to those at sea level. In pre-eclampsia at high altitude, maternal erythropoietin levels remain similar to controls, but lower circulating erythropoietin receptor levels suggest reduced bioavailability [85]. Women with multigenerational high-altitude ancestry (e.g. Andean or Tibetan) are less prone to altitude-related pre-eclampsia than those of lowland descent [75]. Given that adaptive *EPAS1* variants in Tibetans [11,45,46] and Andeans [10–12,46] seem to dampen hypoxia signalling under low-O_2_ conditions, and that *EPAS1* and *PRKAA1* modulate hundreds of downstream HIF/AMPK-α1 targets, it is plausible that blunted HIF coupled with augmented AMPK-α1 activity may prevent aberrant responses to hypoxia that moderate, directly or indirectly, [Hb], maternal health, and placental factors. Moreover, AMPK-α1 activation in mature erythroblasts induces cell cycle arrest in the S phase, autophagy, and caspase-dependent apoptosis, whereas no such effects are observed in similarly treated immature erythroblasts [23]. This is intriguing, because while augmented *PRKAA1* expression and thus AMPK-α1 activity during the final stages of erythropoiesis may ordinarily be deleterious, it also offers a possible further mechanism over and above *EPAS1* pathways for maintaining a relatively low Hct and may thereby offer a further, perhaps crucial, evolutionary advantage for Andean highlanders.

A major challenge in understanding human adaptation is uncovering how interconnected biological factors and traits work together to shape survival and reproductive success. Genetic high-altitude adaptations influence a dynamic network of traits such as haematological, breathing, and vascular responses as well as metabolic regulation. The interplay among these factors probably determines how well individuals cope with hypoxia, with the presence or absence of key adaptations potentially tipping the balance between resilience and risk, which is particularly important in pregnancy, where O₂ supply is critical for fetal development. Beyond high-altitude cohorts, large lowland populations offer valuable opportunities to investigate genetic variants involved in hypoxia responses. By mining public genome datasets and biobanks, researchers can assess whether information regarding high-altitude adaptive variants, genes, or pathways similarly protect against hypoxia-related complications in broader human populations. Such cross-population analyses will deepen our understanding of the genetic mechanisms underlying hypoxia tolerance and could reveal variants with generalizable clinical benefits.

## 5. Conclusions

Our studies offer valuable insights across genetics, respiratory, haematological, metabolic, vascular, and sleep adaptations in adult highlanders and shed light on how these systems support survival in high-altitude environments. We hope these findings will be useful in the context of pregnancy at altitude, complementing ongoing research on maternal and reproductive health. By connecting our work with studies focused on pregnancy, we aim to deepen our understanding of how high-altitude adaptations are passed to offspring and influence fetal development and long-term health outcomes.

## Data Availability

This article has no additional data.
